# Supplementary material for: COVID-19 aerosol transmission simulation-based risk analysis for in-person learning
Source: PLoS One. 2022 Jul 21;17(7):e0271750. doi: 10.1371/journal.pone.0271750 (PMC9302819; doi:10.1371/journal.pone.0271750)
Supplement: S1 Appendix — (ZIP) [file pone.0271750.s001.zip › S1.pdf]

S1 Appendix. Model Equations. We build from the well-mixed, steady-state room model of [1] together with the dose-response model of [2] to estimate the concentration in a given classroom and the likelihood of infection for each individual given the estimated concentration. These two models are reviewed here.

Concentration Model: The aerosol source rate (r_{src} in nL/min from [1]) is: $r_{src} = f_{student} * NI_{student} * (1 - M_{ex}) + f_{faculty} * NI_{faculty} * (1 - M_{ex})$ where $NI_{student}$ and $NI_{faculty}$ are the number of infected students and faculty in the room, M_{ex} is the fraction of aerosol particle blocked from exhaled air by masks if masks are present, and $f_{student}$ and $f_{faculty}$ represent the aerosol rates for students (set to 1 nL/min to indicate minimal talking) and for faculty (set to 5 nL/min to indicate lecturing).

We follow [1] in defining the aerosol decay factor to be $f_a = \tau_a/(\tau_{room} + \tau_a)$. Here $\tau_{room} = 60/ACH$ where ACH is the air changes per hour of the room. τ_a represents other mechanisms through with aerosol particles are removed from the air in a room including settling, deactivation through natural decay, deactivation through interaction with a UVC field (if present) and potentially removal through filtration by an in-room filter. Note that we did not include in-room HEPA filters in our study due to concerns over interference of their noise with teaching as well as the recording of lectures that occurs in many college classrooms. In our case the total decay rate $\tau_a = 1/(1/t_{deact} + 1/t_{settle} + 1/t_{UV})$. t_{deact} was set to 90 minutes and t_{settle} was set to 20 minutes in line with [1]. The effect of UVC on virion deactivation were based on data provided by the manufacturer of a particular type of fan-mounted, up-shining UVC, Big Ass Fans [3, 4]. This data was based on chamber simulation experiments using irradiation susceptibility constants ($k = 0.377 m^2/J$) for live SARS CoV-1 from [5] and inactivation modeling in Equation (1) of [6], in line with design recommendations from the American Society of Heating, Refrigeration, and Air-Conditioning Engineers [7, 8]. This k-value from SARS-CoV-1 is considered conservative (note the larger the k-value, the more susceptible a pathogen is to UVC), as evidence mounts that this value is greater than those found for SARS CoV-2 in liquid and on surfaces [9, 10], and SARS-CoV-2 is demonstrated to be an order of magnitude more susceptible to UVC deactivation when aerosolized [11]. t_{UV} was calculated as $t_{UV} = 1/(k * L * t_{uv})$ with $k = 0.377\ m^2/J$, $L = 1\ \mu\text{W/cm}^2$, and $t_{uv} = 115$ sec. With unit conversions that is $t_{uv} = 1/(0.377 * 115/100) = 2.31$ minutes.

We follow [1] in assuming a viral load of $\rho_0 = 1000$ virions/nL. We also follow [1] in assuming a breathing rate of 10 L/min with a separate mask removal efficiency for inhalation, possibly different from exhalation, of M_{in}. We then define $r_b = 10 * (1 - M_{in})$. For a given room with volume V we define $r_{room} = V/\tau_{room}$

The concentration in the room is then given by $\rho_A = \rho_0 * (r_{src}/r_{room} * f_a)$. The exposure to each individual in the room then given by $exposure = \rho_A * r_b * t_{occupancy}$ where $t_{occupancy}$ is the amount time the individual is the room.

Infection Likelihood Model: Finally, the probability of an individual being infected given a cumulative daily dose of D_{day} is given by $1 - exp(-D_{day}/k$. Because the k value is unknown for SARS-CoV-2, we bounded by the k values for a different coronavirus from [2] and run the model for both k=75 (high transmissibility) and k=500 (low transmissibility).

References

1. Evans M. Avoiding COVID-19: Aerosol Guidelines; 2020. `https://www.medrxiv.org/content/10.1101/2020.05.21.20108894v3.full.pdf`.

2. Watanabe T, Bartrand TA, Weir MH, Omura T, Haas CN. Development of a dose-response model for SARS coronavirus. Risk Anal. 2010;30(7):1129–1138.

3. BIG ASS FANS ION TECHNOLOGY; 2020. `https://cleanairsystem.com/technology/?section=uv-c-technology`.

4. Crist R. Can this smart ceiling fan kill the coronavirus? Independent tests say 'yes'; 2020. `https://www.cnet.com/home/smart-home/can-this-big-ass-fans-haiku-uvc-smart-ceiling-fan-kill-covid-19-independent-tests-say-yes-coronavirus/`.

5. Kowalski W. Ultraviolet germicidal irradiation handbook: UVGI for air and surface disinfection. Springer science & business media; 2010.

6. Kowalski W, Bahnfleth WP, Witham D, Severin B, Whittam T. Mathematical modeling of ultraviolet germicidal irradiation for air disinfection. Quantitative microbiology. 2000;2(3):249–270.

7. Stewart EJ, Schoen LJ, Mead K, Olmsted RN, Sekhar C, Vernon W, et al. ASHRAE position document on infectious aerosols. ASHRAE: Atlanta, GA, USA. 2020;.

8. Ultraviolet air and surface treatment. In: Proc. ASHRAE Handbook-HVAC Appl.; 2019. p. 1–18.

9. Biasin M, Bianco A, Pareschi G, Cavalleri A, Cavatorta C, Fenizia C, et al. UV-C irradiation is highly effective in inactivating SARS-CoV-2 replication. Scientific Reports. 2021;11(1):1–7.

10. Storm N, McKay LG, Downs SN, Johnson RI, Birru D, de Samber M, et al. Rapid and complete inactivation of SARS-CoV-2 by ultraviolet-C irradiation. Scientific Reports. 2020;10(1):1–5.

11. Beggs CB, Avital EJ. Upper-room ultraviolet air disinfection might help to reduce COVID-19 transmission in buildings: a feasibility study. PeerJ. 2020;8:e10196.
