# Supplementary material for: COVID-19 aerosol transmission simulation-based risk analysis for in-person learning
Source: PLoS One. 2022 Jul 21;17(7):e0271750. doi: 10.1371/journal.pone.0271750 (PMC9302819; doi:10.1371/journal.pone.0271750)
Supplement: S2 Appendix — (ZIP) [file pone.0271750.s002.zip › S2.pdf]

S2 Appendix. Algorithm Structure.

Algorithm 1 Class Period Exposure

```
if infected students = 0 then
    Continue to next class
else
    factors ← (room height, width, airflow, employed mitigation measures, class duration, infected students)
    x ← ExposureDose(factors)
    Get list of infectable students
    Increment infectable students' viral exposure by x
end if
```

Algorithm 2 Daily Progression

```
for class in classes do
    Run Class Period Exposure algorithm
end for
for student in students do
    Evaluate student's exposure for day in dose-response function
    if student becomes infected then
        Get infectivity duration from equation X
    else if student does not become infected then
        Reset student's exposure to 0 for next day
    end if
end for
```

Algorithm 3 Semester Progression

```
for i in 13 weeks do
    for j in 7 days do
        if j is weekday then
            Run Daily Progression algorithm
        end if
        for student in infected students do
            Decrement student's remaining infectious days by 1
            if Days since infection = 2 then
                Remove from class until recovered
            end if
            if student's remaining infectious days = 0 then
                Add student to immune list
                Add student to previously-infected list
            end if
        end for
    end for
end for
for student in previously-infected list do
    Evaluate student's health outcomes with probability distribution X
    Increment appropriate count for given health outcome
end for
```
