# Supplementary material for: COVID-19 aerosol transmission simulation-based risk analysis for in-person learning
Source: PLoS One. 2022 Jul 21;17(7):e0271750. doi: 10.1371/journal.pone.0271750 (PMC9302819; doi:10.1371/journal.pone.0271750)

S3 Appendix. Inverse cumulative density functions for various outcomes in the college under the different scenarios. Each graph shows the curve of the probability of exceeding a given number of infections, hospitalizations, or deaths.

Fig 1. Inverse cumulative density functions for total number of students infected in the college.

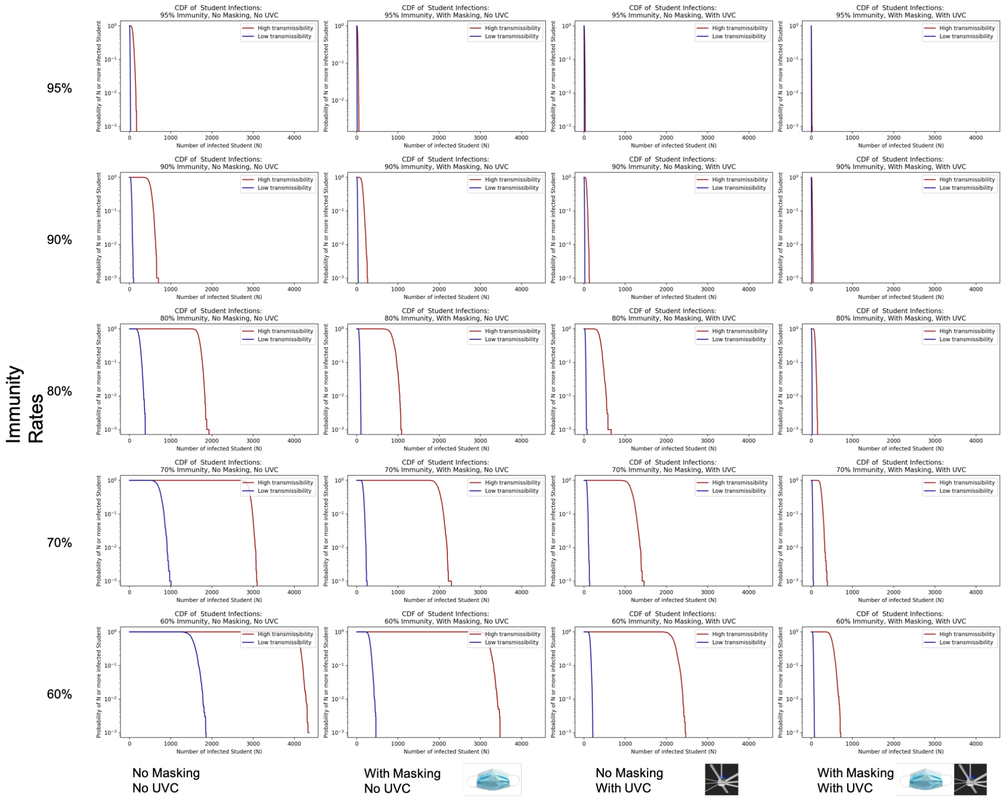

Fig 2. Inverse cumulative density functions for total number of faculty infected in the college.

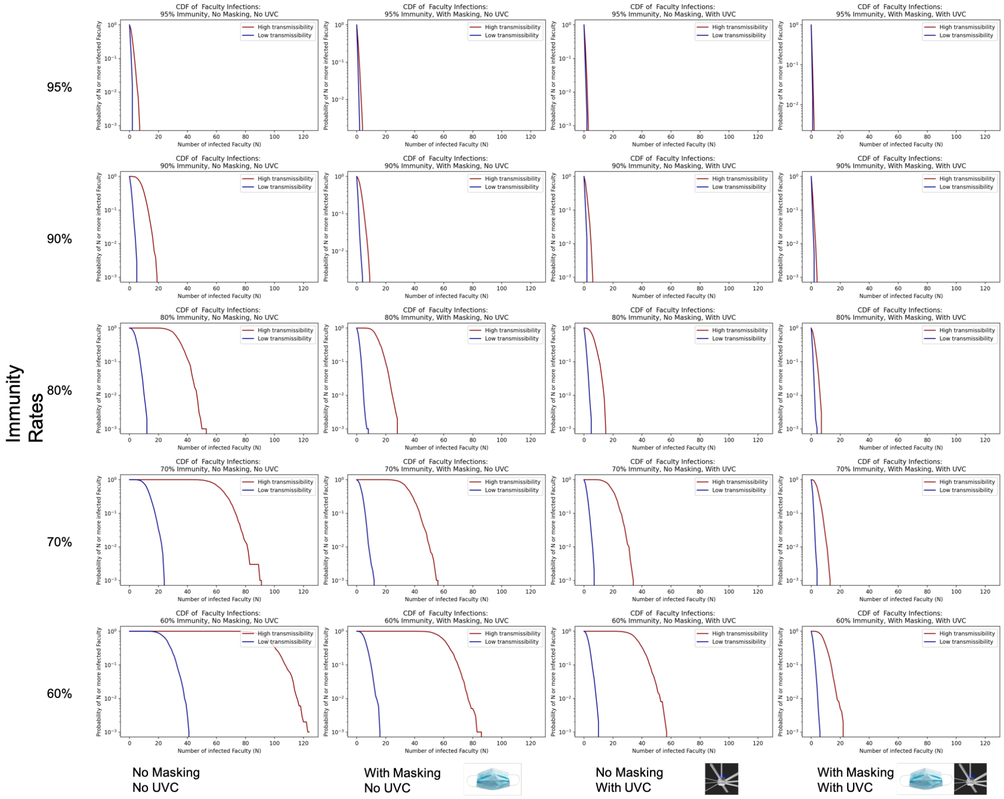

Fig 3. Inverse cumulative density functions for total number of student hospitalizations in the college.

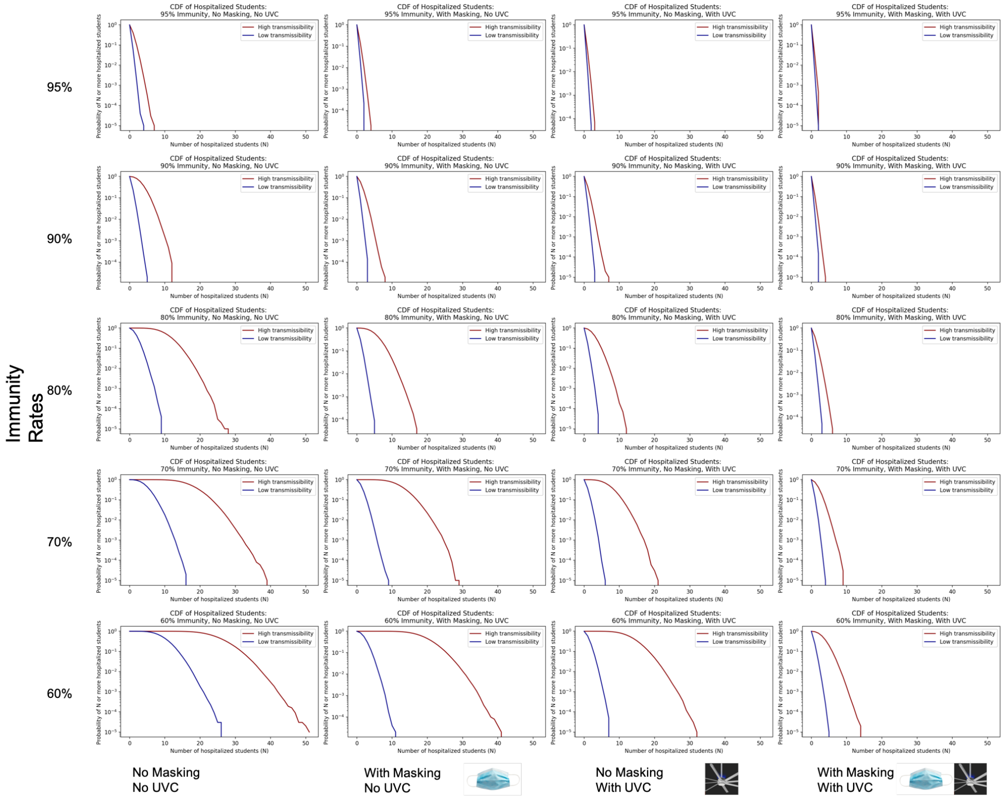

Fig 4. Inverse cumulative density functions for total number of faculty hospitalizations in the college.

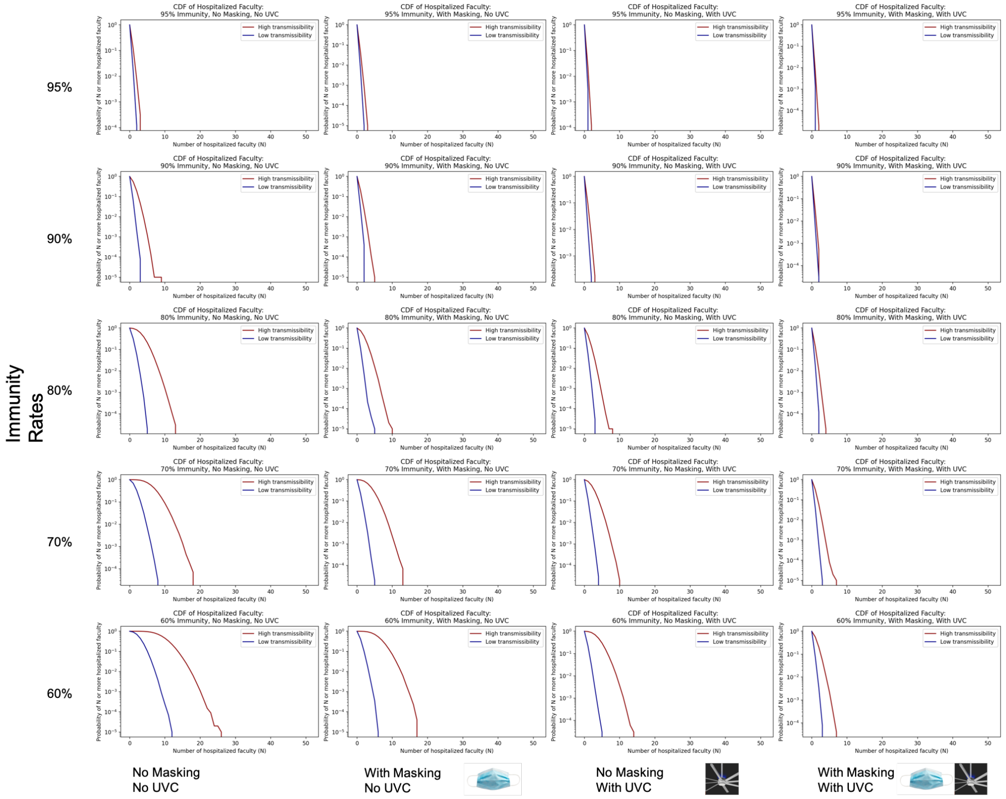

Fig 5. Inverse cumulative density functions for total number of student deaths in the college.

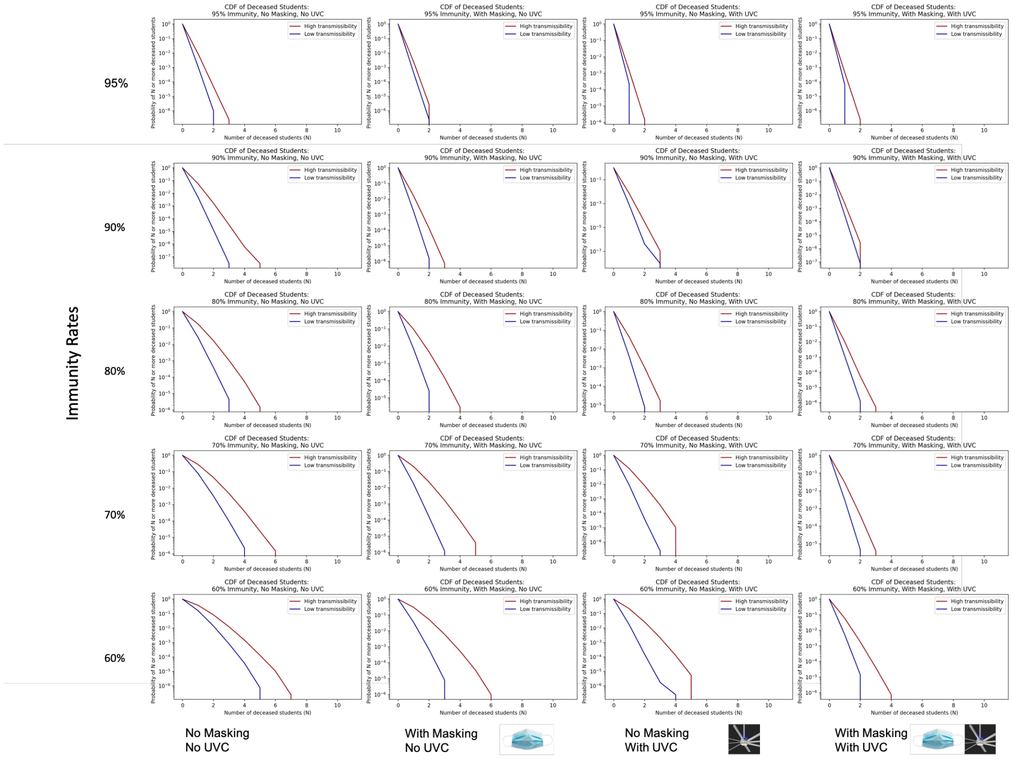

Fig 6. Inverse cumulative density functions for total number of faculty deaths in the college.

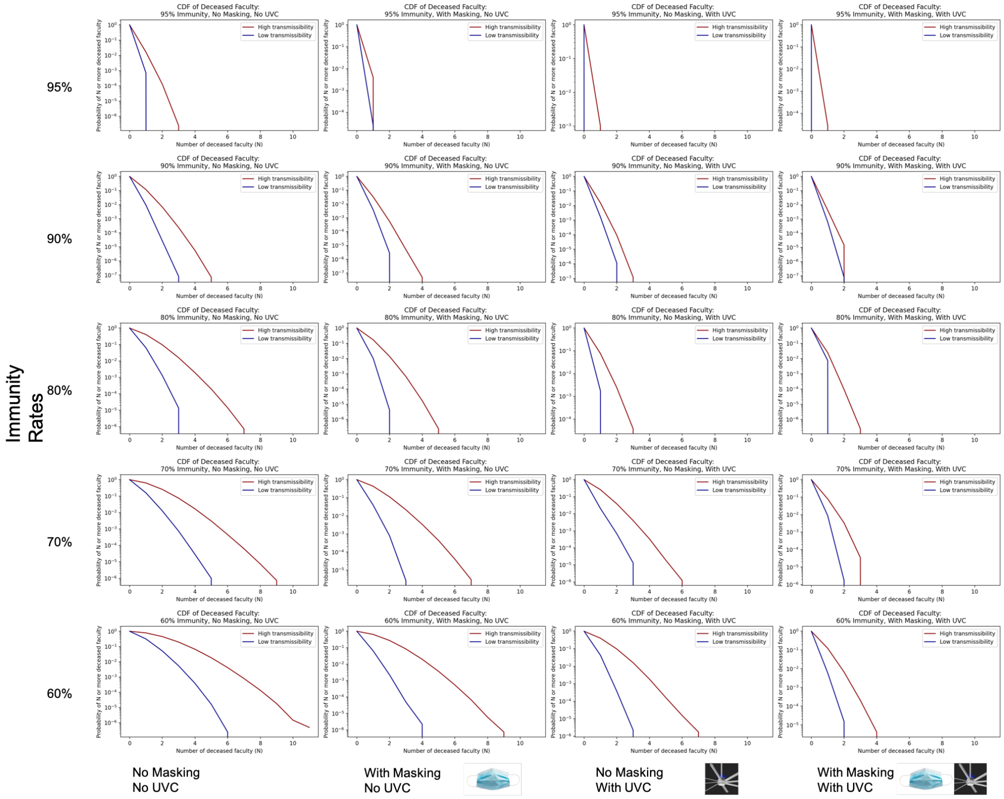

Supplement: S3 Appendix — Each graph shows the curve of the probability of exceeding a given number of infections, hospitalizations, or deaths. (ZIP) [file pone.0271750.s003.zip › S3.pdf]
