# Supplementary material for: COVID-19 aerosol transmission simulation-based risk analysis for in-person learning
Source: PLoS One. 2022 Jul 21;17(7):e0271750. doi: 10.1371/journal.pone.0271750 (PMC9302819; doi:10.1371/journal.pone.0271750)

S4 Appendix. Individual Probability of Outcomes by Immunity Rates and Age Group.

Fig 1. Cumulative density functions over the probabilities of individual student and faculty infection given 60% immunity in the college.

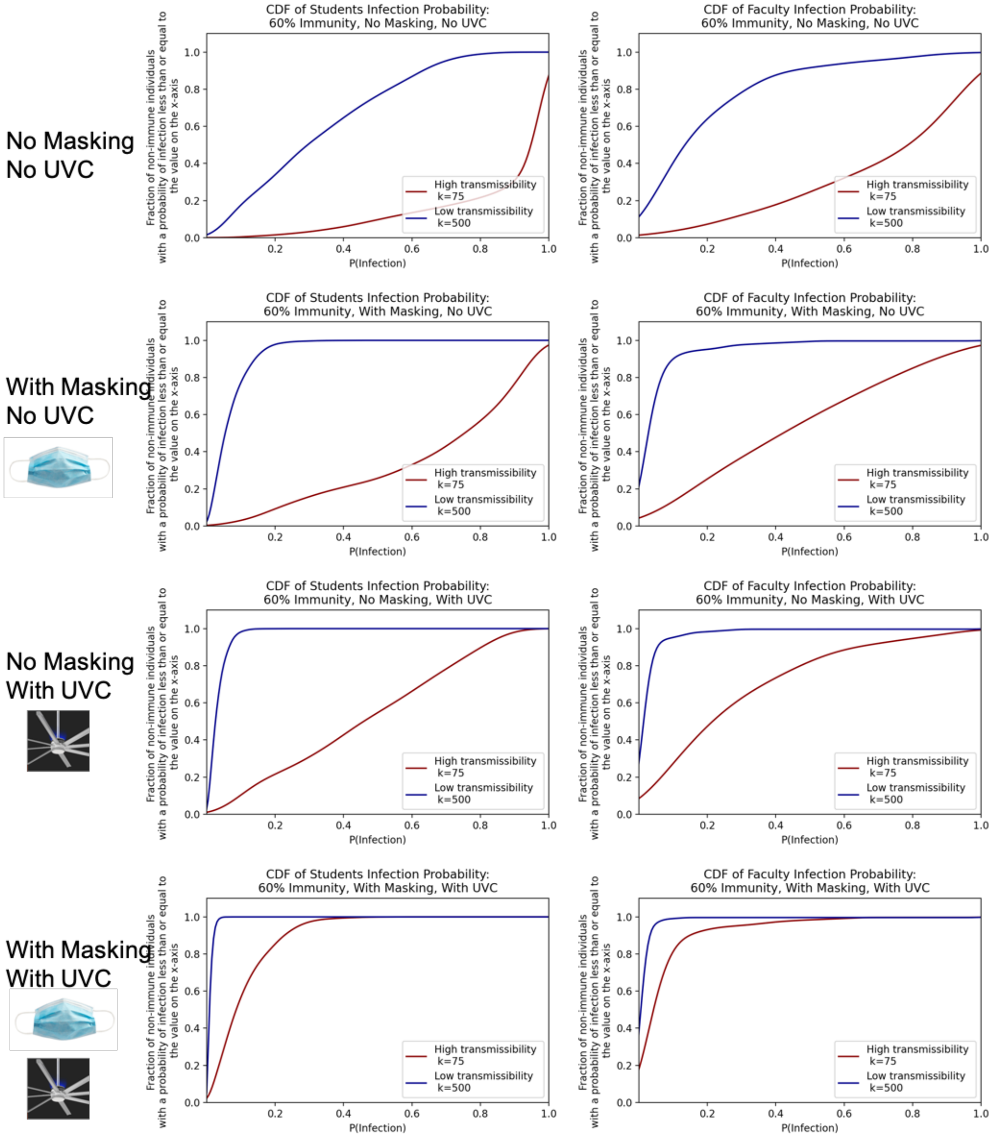

Fig 2. Cumulative density function for the probability of hospitalization by age group given 60% immunity. Note that all students are lumped together into one age group, assumed to be the 18-24 age group.

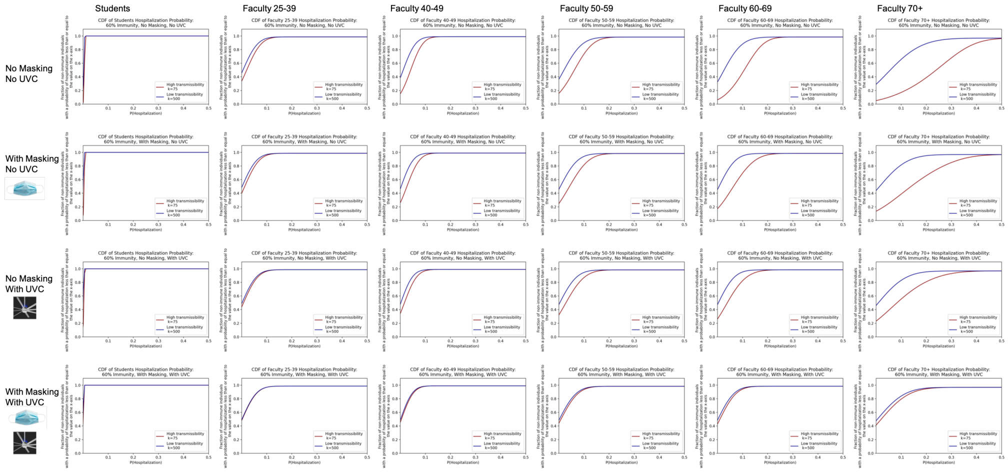

Fig 3. Cumulative density function for the probability of death by age group given 60% immunity. Note that all students are lumped together into one age group, assumed to be the 18-24 age group.

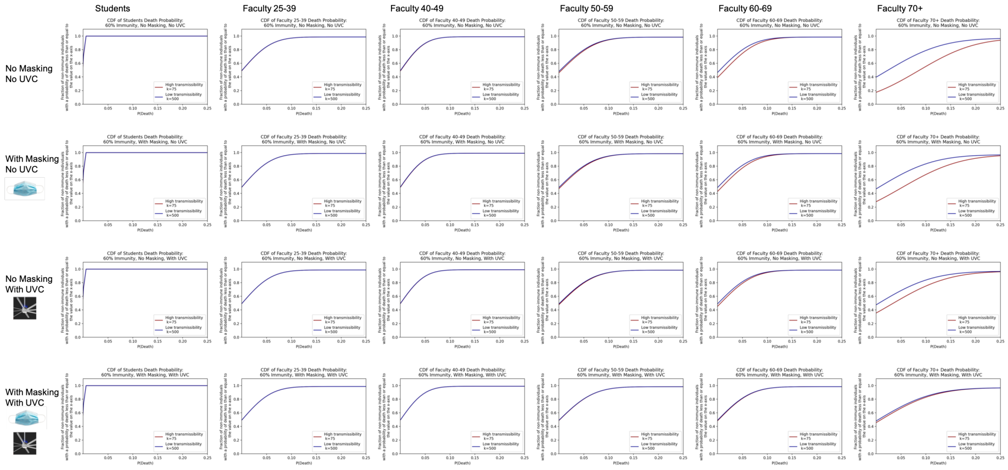

Fig 4. Cumulative density functions over the probabilities of individual student and faculty infection given 70% immunity in the college.

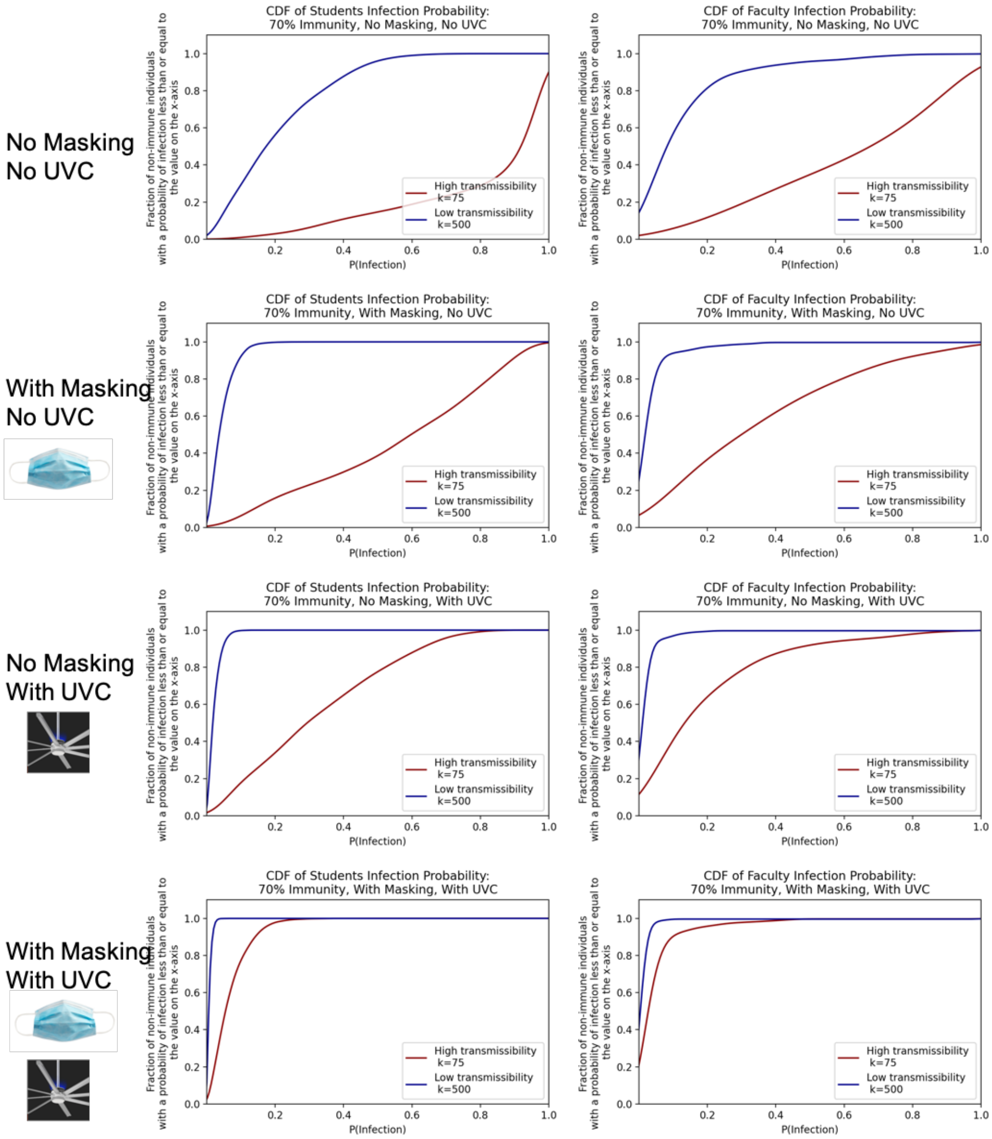

Fig 5. Cumulative density function for the probability of hospitalization by age group given 70% immunity. Note that all students are lumped together into one age group, assumed to be the 18-24 age group.

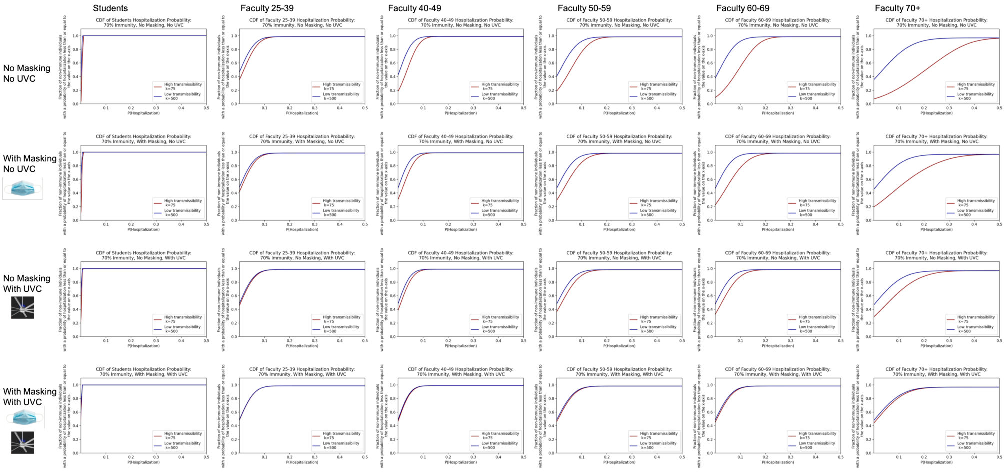

Fig 6. Cumulative density function for the probability of death by age group given 70% immunity. Note that all students are lumped together into one age group, assumed to be the 18-24 age group.

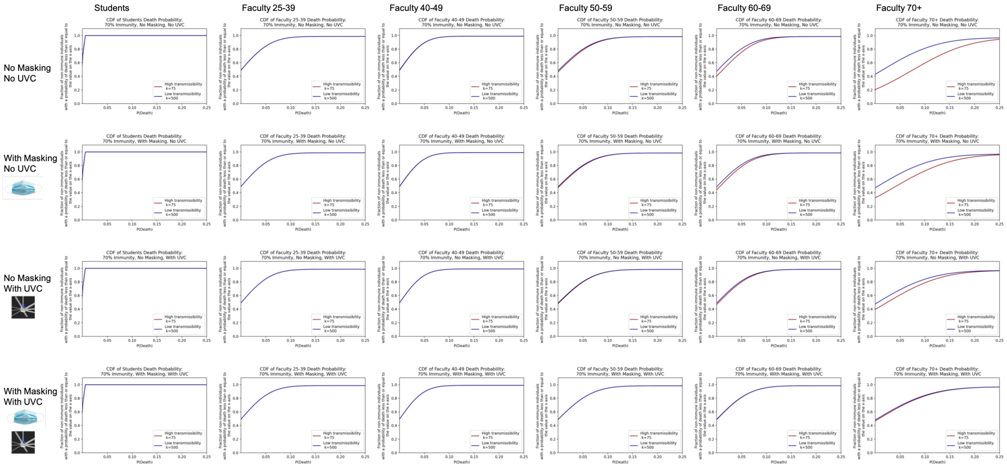

Fig 7. Cumulative density functions over the probabilities of individual student and faculty infection given 80% immunity in the college.

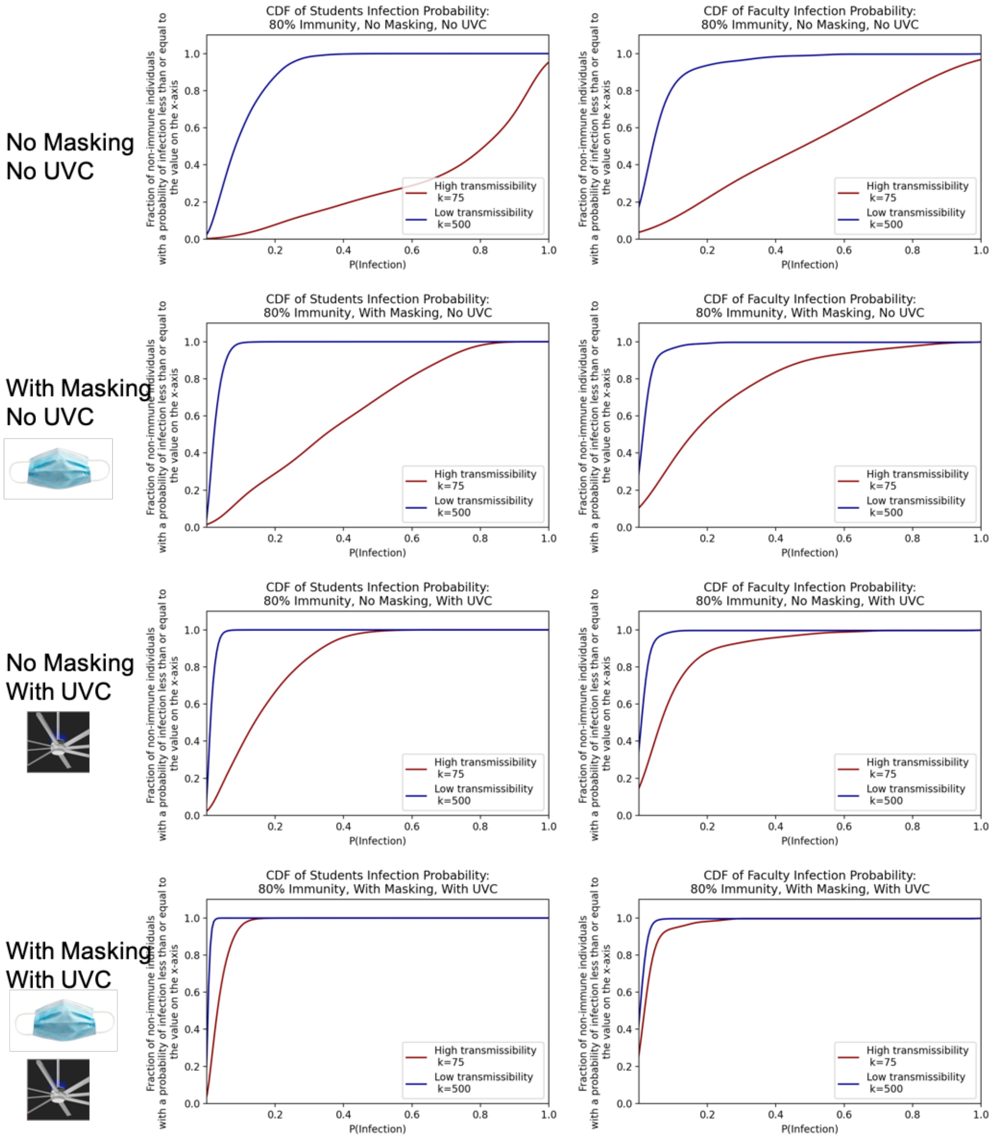

Fig 8. Cumulative density function for the probability of hospitalization by age group given 80% immunity. Note that all students are lumped together into one age group, assumed to be the 18-24 age group.

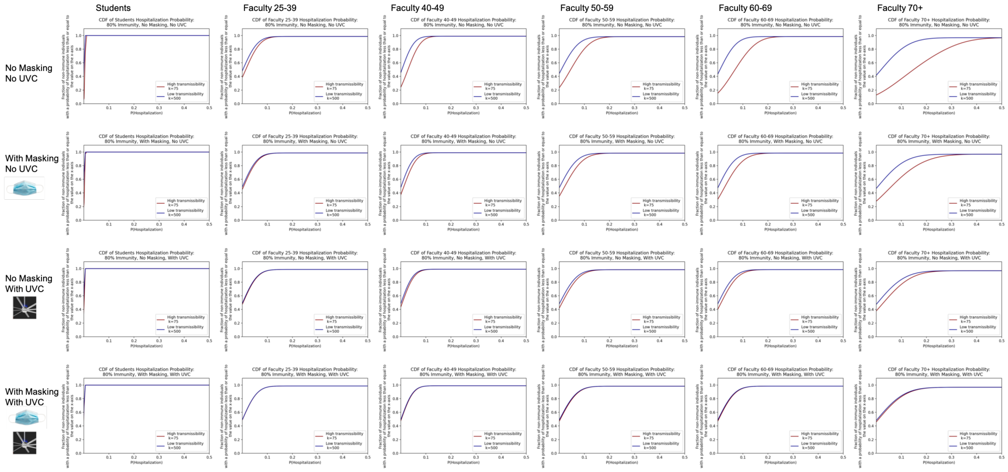

Fig 9. Cumulative density function for the probability of death by age group given 80% immunity. Note that all students are lumped together into one age group, assumed to be the 18-24 age group.

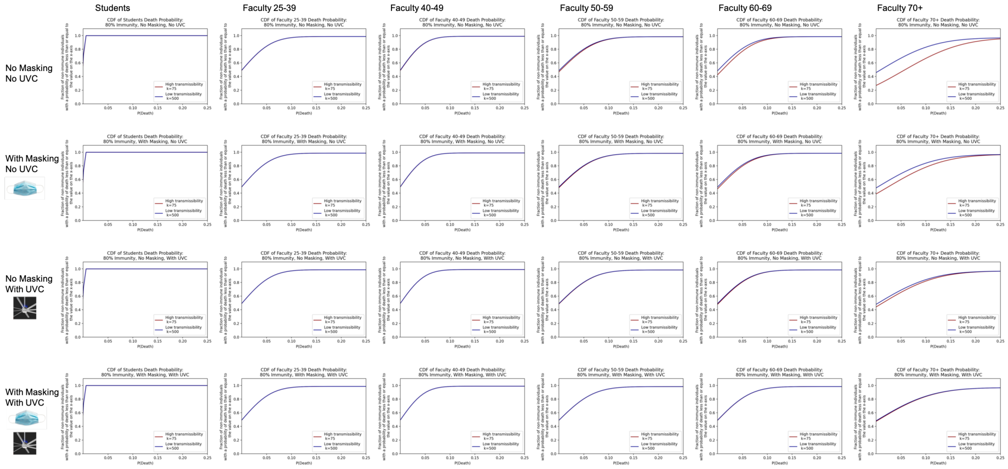

Fig 10. Cumulative density functions over the probabilities of individual student and faculty infection given 90% immunity in the college.

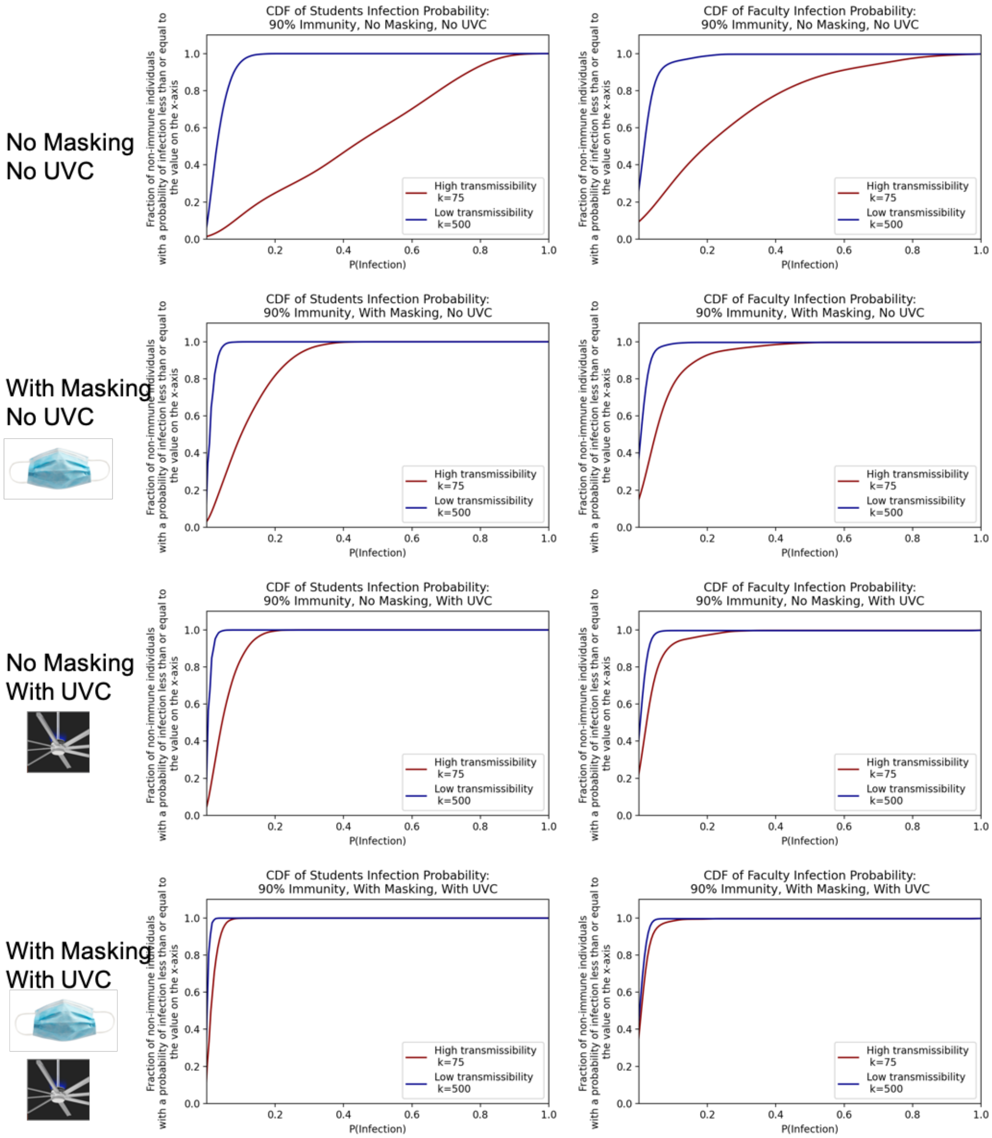

Fig 11. Cumulative density function for the probability of hospitalization by age group given 90% immunity. Note that all students are lumped together into one age group, assumed to be the 18-24 age group.

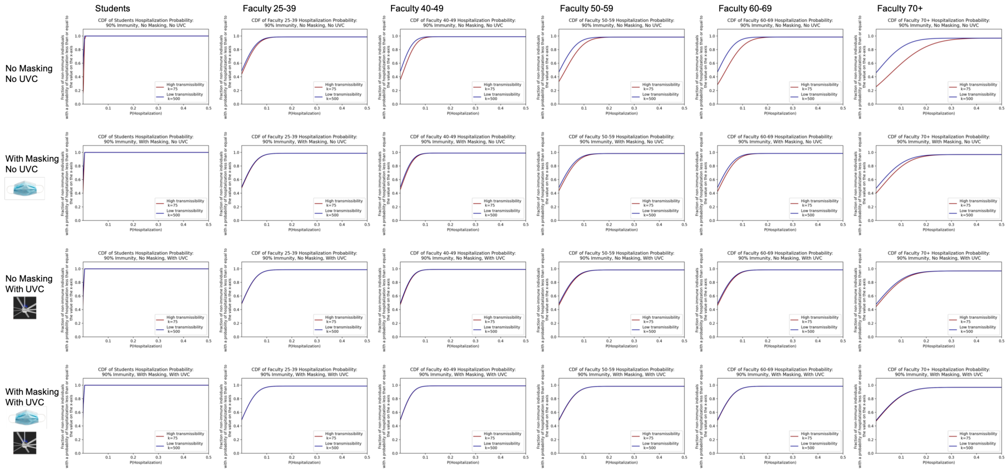

Fig 12. Cumulative density function for the probability of death by age group given 90% immunity. Note that all students are lumped together into one age group, assumed to be the 18-24 age group.

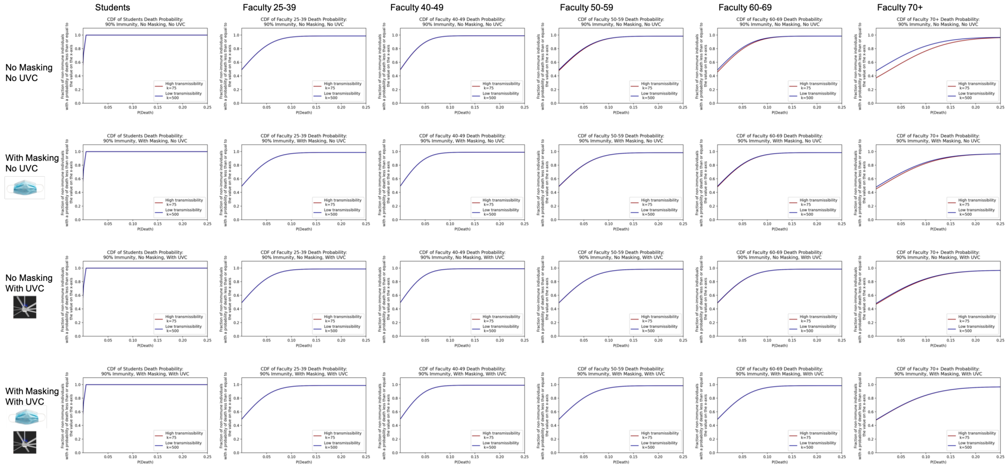

Fig 13. Cumulative density functions over the probabilities of individual student and faculty infection given 95% immunity in the college.

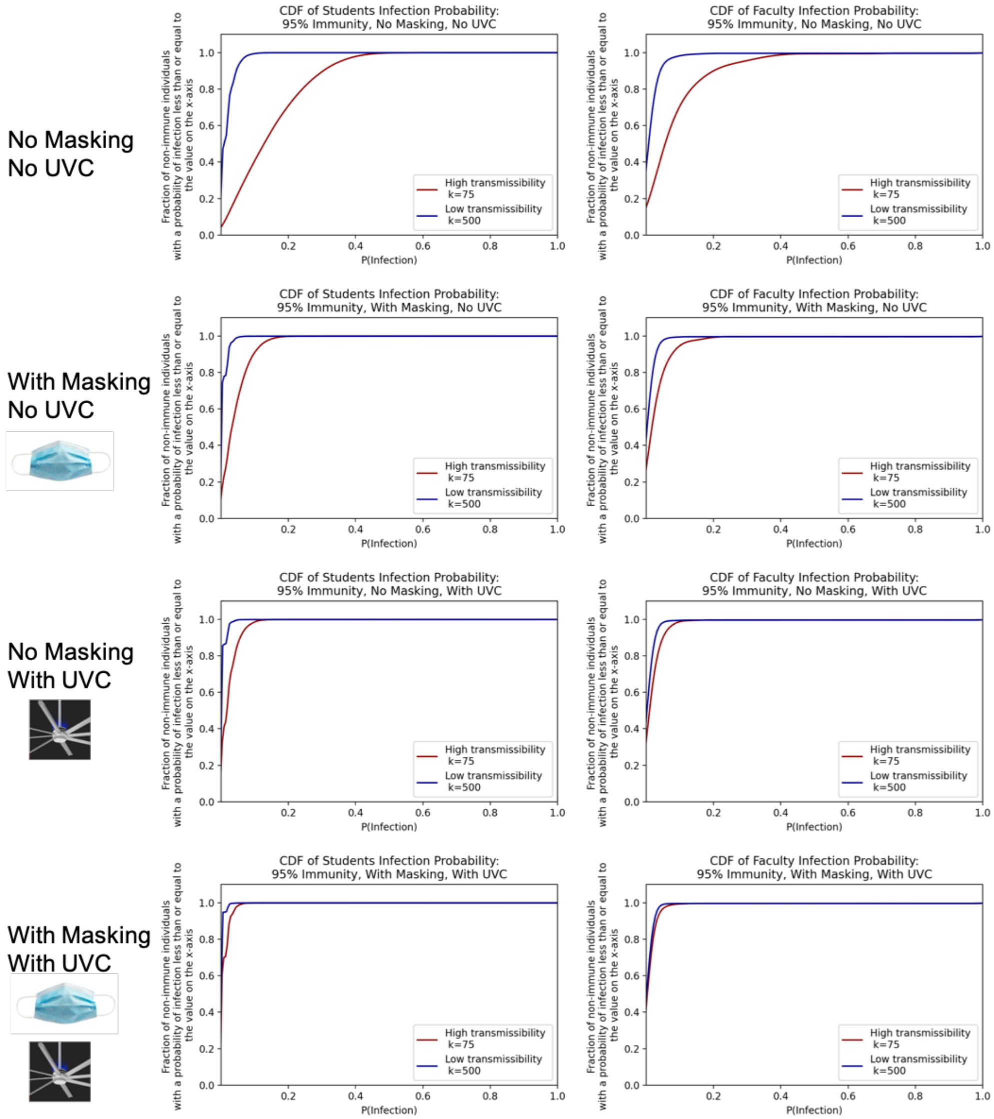

Fig 14. Cumulative density function for the probability of hospitalization by age group given 95% immunity. Note that all students are lumped together into one age group, assumed to be the 18-24 age group.

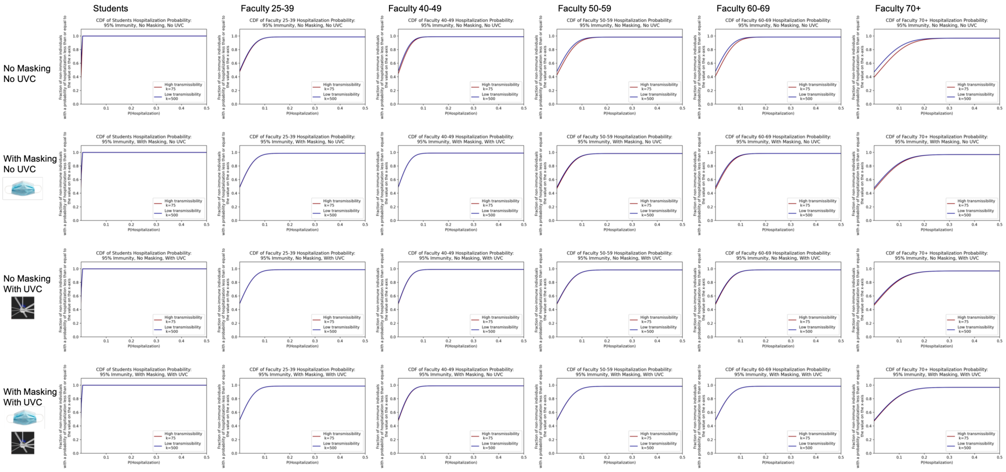

Fig 15. Cumulative density function for the probability of death by age group given 95% immunity. Note that all students are lumped together into one age group, assumed to be the 18-24 age group.

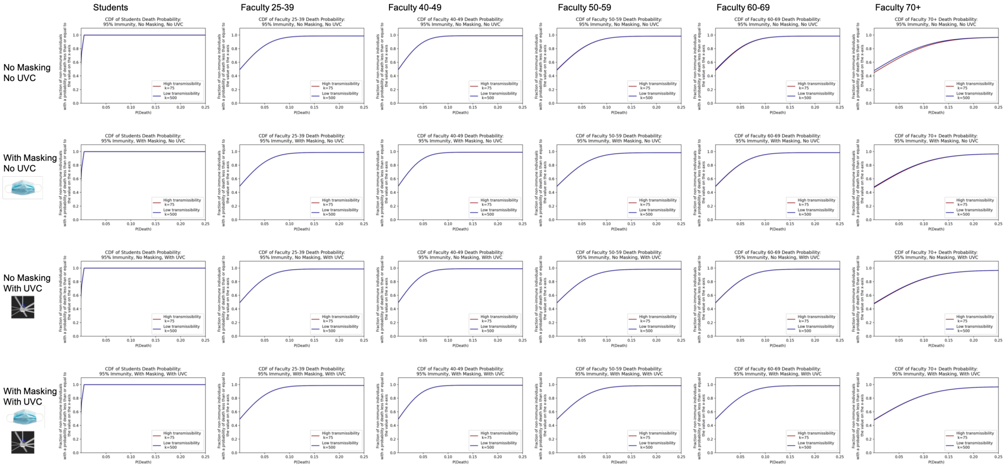

Supplement: S4 Appendix — (ZIP) [file pone.0271750.s004.zip › S4.pdf]
