# Supplementary material for: COVID-19 aerosol transmission simulation-based risk analysis for in-person learning
Source: PLoS One. 2022 Jul 21;17(7):e0271750. doi: 10.1371/journal.pone.0271750 (PMC9302819; doi:10.1371/journal.pone.0271750)
Supplement: Attachment — Submitted filename: Response to Reviewers_rev2.pdf [file pone.0271750.s006.pdf]

To our reviewers,

Thank you so much for your time in thoughtfulness in reviewing our manuscript “COVID-19 aerosol transmission simulation-based risk analysis for in-person learning.” We respond to the review of our last submission below:

Reviewer #2: I appreciate the authors' response to my comments, and I am particularly happy that they consider infection endogenously in their model. The background section of the paper can be further improved (still citation issues, and in many places, you can re-write to avoid listing names of authors of papers that are only marginally related to your work). Otherwise it looks fine to me.

Just for the authors' information, I would like to write that SEIR is referred to any model that categorizes people in these groups, but that doesn't mean that an agent-based model is not an SEIR. In your writing it felt like you use SEIR to refer to "Compartmental" models (yes, all compartmental models of infection are SEIR-like models), and then you have a different category for agent-based model. A more accurate categorization of SEIR models is compartmental models vs. agent-based models. This is very minor issue and a common mistake in the modeling community. Good luck, and thanks for sharing your paper with me.

> We rewrote a portion of our Background section (starting with line 117) to better clarify the differences between agent-based models, compartmental models, and our model presented in this manuscript. We also more concisely summarized the literature corresponding to those models and model differences.

Additionally, we have updated the following citations to reflect current publication status:

> Rosenstrom E, Ivy J, Mayorga M, Swann J, Oruc BE, Keskinocak P, et al. High-quality masks reduce covid-19 infections and death in the US. In: 2021 Winter Simulation Conference (WSC). IEEE; 2021. p. 1–11.
>
> Ranoa DRE, Holland RL, Alnaji FG, Green KJ, Wang L, Fredrickson RL, et al. Mitigation of SARS-CoV-2 transmission at a large public university. Nature communications. 2022;13(1):1–16.
